# Shaping the RF Transmit Field in 7T MRI Using a Nonuniform Metasurface Constructed of Short Conducting Strips

**DOI:** 10.1021/acsami.4c10402

**Published:** 2024-08-31

**Authors:** Santosh Kumar Maurya, Rita Schmidt

**Affiliations:** † Department of Brain Sciences, Weizmann Institute of Science, Rehovot 7610001, Israel; ‡ The Azrieli National Institute for Human Brain Imaging and Research, Weizmann Institute of Science, Rehovot 7610001, Israel

**Keywords:** metasurface, magnetic resonance imaging, ultrahigh field MRI, RF transmit enhancement, brain imaging

## Abstract

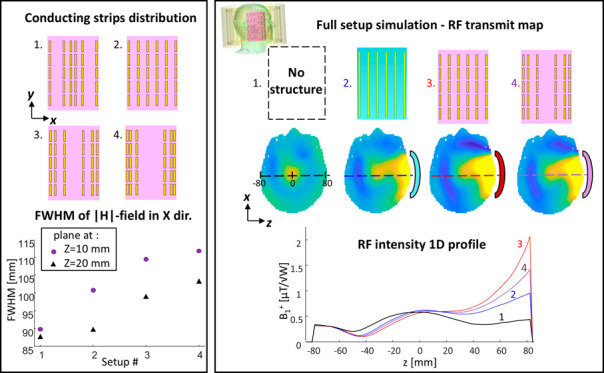

The ability of metamaterial structures to offer unique properties and new solutions has opened new avenues in a wide range of applications, including super-resolution in optics and efficient antennas in radiofrequency (RF) engineering. In magnetic resonance imaging (MRI), metamaterials hold the promise of increasing the RF magnetic field intensity while minimizing power deposition. Here, we propose a metasurface based on a two-dimensional (2D) array of short conducting strips combined with a high dielectric substrate, which was tuned to operate at ultrahigh field 7T human MRI. While studied in optics and electromagnetics in the GHz-to-THz range, this study is the first to design such a metasurface for proton imaging at 7T MRI. We performed electromagnetic (EM) simulation of the brain MRI setup with the new metasurface placed in the proximity to the temporal lobe, which showed 2.2-fold local increase in the RF transmit efficiency, with superior performance than an array of electric dipoles. In this study, we also investigate the effect of the spatial distribution of the subunits to control the target RF field’s distribution. While the common design is based on a uniform distribution of the subunits, nonuniform distribution, such as a denser center (convex) or more condensed edges (concave), provides an extra dimension to tailor both the magnetic and electric fields. The concave distribution achieved 1.5–1.8-fold reduction in the power deposition compared to the uniform distribution in the brain MRI setups examined.

## Introduction

As part of the effort to increase the resolution of ultrahigh field (≥7 T) magnetic resonance imaging (MRI) scanners, for both research and better diagnostics purposes, growing attention has been given to improving these system’s radiofrequency (RF) coils. Traditional RF coils in MRI commonly feature a symmetric arrangement of capacitive and inductive elements including geometries based on volume coil designs (e.g., birdcage and TEM coils) and local or surface coils (e.g., loops and dipole antennas). Inspired by metamaterial innovations in the optical (tens-to-hundreds of THz) and microwave (tens-to-hundreds of GHz) frequency ranges, possible translations to MRI RF frequencies have been tested. These include examinations of setups with various subunits, including structures based on magnetic dipoles such as magneto-inductive lens,^1^ designs based on capacitively loaded metallic rings^2^ and metallic helices,^3^ and ones based on electric dipoles such as arrays of metallic wires.^4−6^ However, a major drawback in many of these implementations is that they require large unit-cells or a high number of lumped elements. Alongside the need to overcome this limitation, improved RF resonator designs should also increase the RF transmit efficiency (maximizing the H-field) and offer the ability to tailor and reduce the power deposition as well as the local hotspots of the electric field.

An accessible approach to reduce the size of the unit-cells is a hybrid design that combines a high permittivity dielectric layer and a set of conductors.^7−11^ Such a setup would offer practicable resonant modes with deep enough penetration for MRI applications. Another advantage is that the structures of the hybrid design would be much thinner than those of implementations based only on a dielectric layer. Here we report on the design of such an RF resonator tuned at 298 MHz for imaging at 7T MRI. Our design is based on a two-dimensional (2D) array of short conducting strips combined with a high dielectric substrate. This type of assembly has already been well studied in optics’ and electromagnetics’ GHz-to-THz range,^12−14^ but here we examine, for the first time, such assembly specifically designed for proton imaging in MRI.

A comparative analysis of the new design to an array of long strips combined with high dielectric layer (which was studied before^9^) is performed. Each design was built while tuning the resonance frequency of the lowest transverse-electric mode to 298 MHz and keeping the overall dimensions.

We further explored the effect of the spatial distribution of the metasurface’s subunits on the resulting electromagnetic (EM) fields. The results were used to optimize the design and leverage the novel capabilities in MRI applications. Studies that utilized a nonuniform geometry of the metamaterials to control the applied transformation were introduced in optics^15−18^ and microwave,^19^ but were not studied for MRI applications. In a recent study,^20^ we showed that a nonuniform distribution of the subunits in an array of electric dipoles can add additional control to tailor the RF field distribution in MRI. Specifically, we found that the spatial distribution of the subunits can play a key role in reducing the local E-field, thus increasing the safety, which is crucial for diagnostic devices. Here, we utilize this approach by distributing the short conducting strips of the proposed design in a nonuniform manner to reduce the power deposition.

In this work, EM simulations were performed for a full (RF related) MRI brain imaging setup (including a birdcage coil for RF transmission) using a virtual human model with the proposed metasurface placed in the proximity of the right temporal lobe. At 7T MRI, the left and right temporal lobes in the brain greatly suffer from significant drop in the RF transmit field intensity, *B*_1_^+^.^21^ This *B*_1_^+^, defined as (*B*_1*x*_ + *iB*_1*y*_)/2, is the MRI RF magnetic field component—describing the left circularly polarized field transverse to the MRI’s main static magnetic field *B*__0__ (along *z*)—which plays a role in the spins’ “excitation”. In MRI, the imaging signal is proportional to the sine of the excitation angle (sin α), where the excitation angle at each point is, in turn, proportional to *B*_1_^+^ at that point. At 7T MRI, the approximation that *B*_1_^+^ is uniform (used at lower magnetic fields) no longer holds since the EM wavelength within the body is no longer larger than the region being imaged. This leads to RF field inhomogeneity that affects the imaging at 7T (and higher fields). A passively added metasurface can be utilized to modify the local *B*_1_^+^, either to improve the RF field homogeneity within the brain or to locally improve signal for targeted imaging applications. Here, we examined local *B*_1_^+^ enhancement in the temporal and occipital lobes achieved with a resonant metasurface with human simulation and experimentally using a phantom that mimics the brain’s electrical properties.

## Results

### Magnetic-Dipole vs Electric-Dipole Design

Several works already examined structures based on a high permittivity dielectric layer and an array of long conducting strips. This design is based on electric dipoles and have similar properties to a metamaterial with negative permittivity,^21^ as was demonstrated in optics. In MRI, it was further demonstrated to provide local RF transmit field enhancement, including in brain imaging and spectroscopy. By replacing the array of conducting strips with a 2D array of short conducting strips, which is based on magnetic dipoles, the similarity to negative permittivity can be replaced with a similarity to negative permeability.^22,23^ Here, we demonstrate and characterize an assembly comprised of a 2D array of short strips tuned at 298 MHz (the Larmor frequency for proton imaging in MRI) that can generate an applicable resonant mode for RF transmission in MRI. We examined the assembly at the lowest transverse-electric (TE) mode since it provides H-field components that are perpendicular to the main static magnetic field and have large penetration depth. Importantly, it is also the mode at which a minimal electric field in the subject (or external to the structure) is achieved.

We selected a setup with overall dimensions of 16 × 11 cm^2^, as it is a practical setup in human MRI brain imaging. The width of 11 cm was chosen to optimally cover the brain in the right–left or anterior–posterior directions, while the length of 16 cm was chosen to easily fit along the head–feet direction within the RF head coil with an inner depth of 18 cm. The TE_01_ mode was achieved with a matrix of 6 × 5 short copper strips of 20 mm in length with a dielectric substrate of relative permittivity (ε_r_) of 164 and a thickness of 7 mm (see detailed description in the Experimental Section). The copper strips’ length—which is a factor of 2 smaller than half the wavelength (∼8 cm, taking the permittivity into account)—and the gaps size were found in simulations to provide a dominant magnetic-dipole behavior rather than an electric-dipole one. Supporting Information, Figure S1 shows the vector plot of the surface currents generated in the designed setup. The required permittivity of 164 can be achieved with a suspension comprised of water and CaTiO_3_ or BaTiO_3_ powders, as was previously demonstrated with dielectric pads in MRI.^24^ In the case that different dimensions are required, a metasurface with other dimensions can be designed to provide a TE_01_ mode at 298 MHz. Supporting Information, Figure S2 shows two structures designed for two additional setups: one with smaller dimensions and one with larger ones.

We compared this design to a metamaterial-based design with an array of six long strips with the same outer dimensions. The long-strip design was also tuned to be resonant at 298 MHz using a dielectric substrate with ε_r_ = 72. In both setups, the strips were equidistantly spaced 20 mm apart.

**Figure 1:**
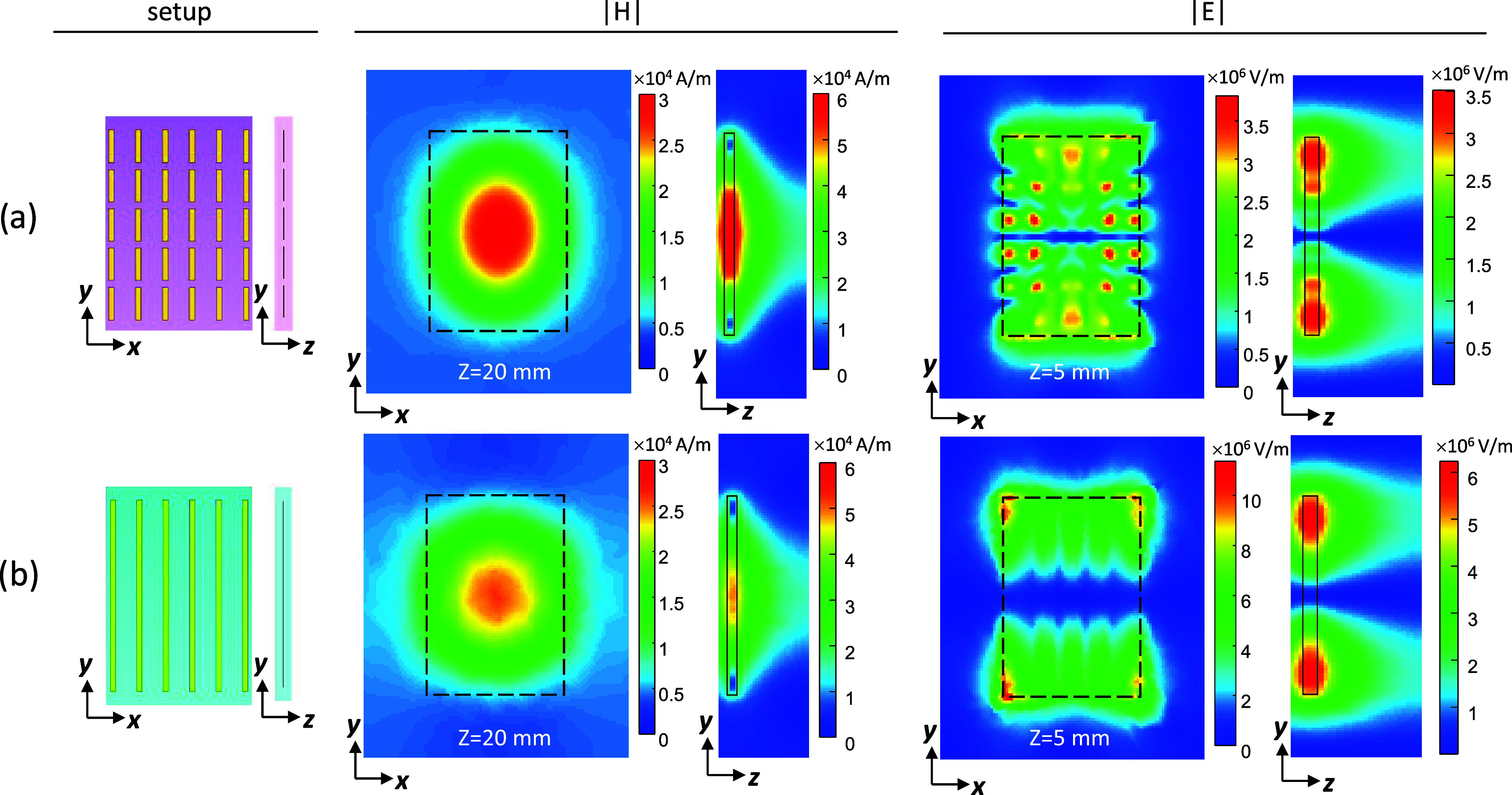
Short- and long-strip resonant structure designs with high dielectric layer. (a) Short-strips. (b) Long-strips. From left to right: The structure schematics and RF field images in two cross sections: the |*H*| field map in parallel to the structure plane located 20 mm from the structure‘s center and in a plane perpendicular to the structure; the |*E*| field map at 5 mm from the structure and in a plane perpendicular to the structure. Black, dashed overlay shows the structure’s dimensions. The short-strips’ structure included a dielectric layer of ε_r_ = 164 and the long-strips’ structure included a dielectric layer of ε_r_ = 72.

**Figure 2:**
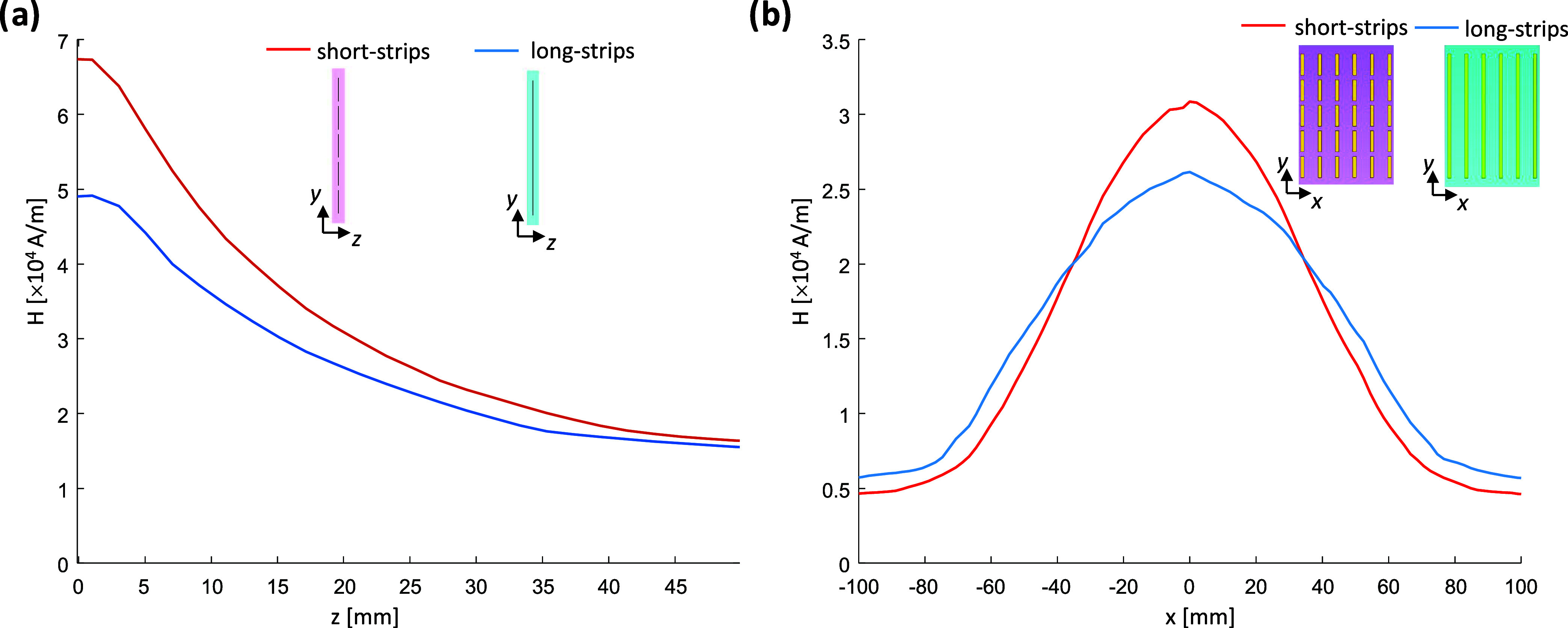
1D profiles of the |*H*| fields (a) perpendicular to the structure at the center and (b) in parallel to the resonant structure in the *X* direction, 20 mm from the structure’s center.

An Eigen-mode solver was used to characterize the H- and E-field distributions of the TE_01_ mode in the short- and long-strip configurations. Figures 1 and 2 show the resulting RF field distributions, as well as one-dimensional (1D) profiles. The |*H*|-field maps are shown at 20 mm away from the structure’s center to represent a case of an imaging slice, while the |*E*|-field maps at 5 mm from the structure’s center (in close proximity to the structure) present the E-field region with the highest intensity. The short-strip metasurface showed a 1.37-fold higher maximum of the H-field compared to the long-strip structure. The maximum of the E-field was 3-fold lower in the short-strip configuration. The actual effect on the power deposition and potential heating is evaluated based on a full setup in a section below.

### Nonuniform Distribution of the Conducting Strips

**Figure 3:**
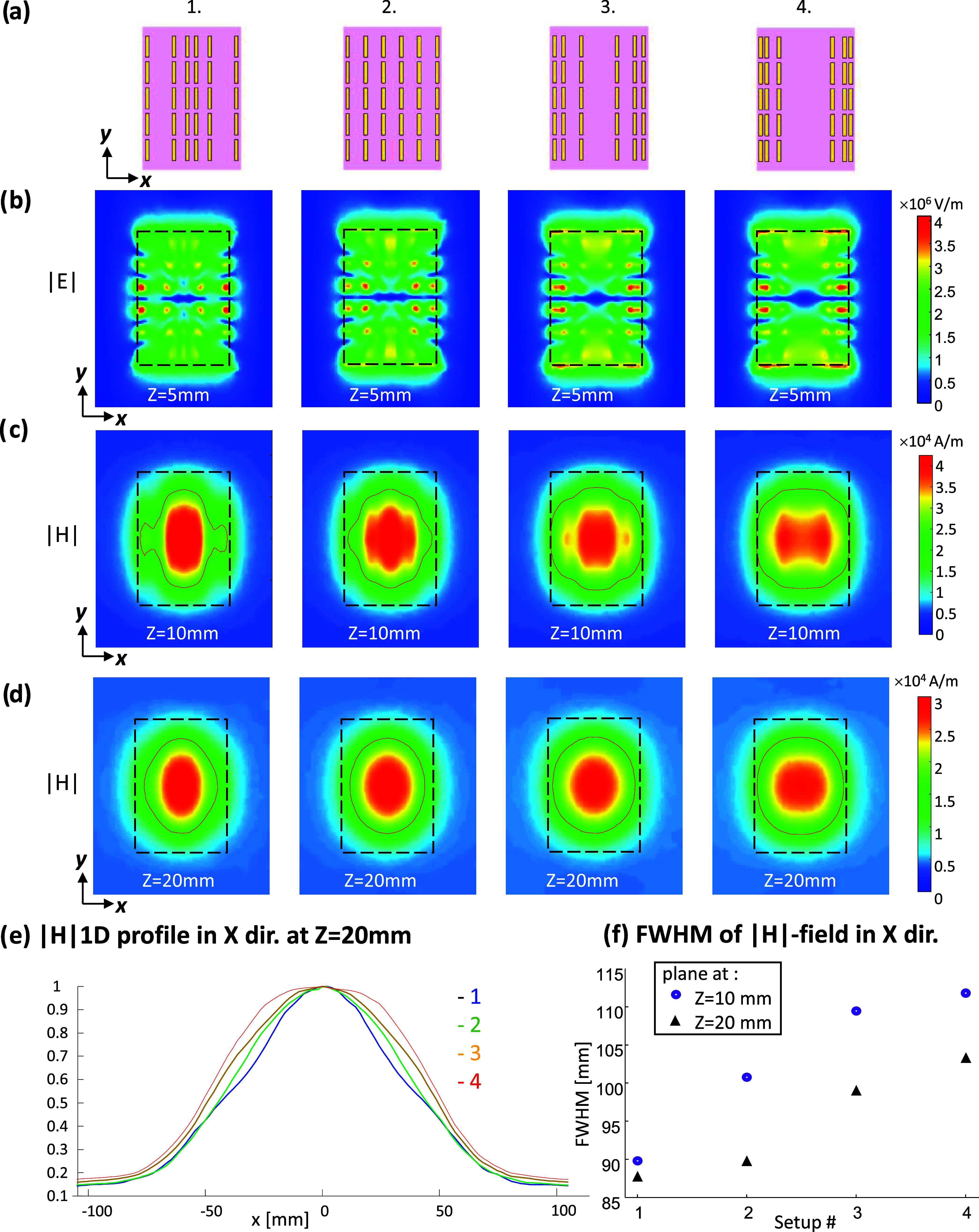
Comparison of the uniform and nonuniform distribution of the strips in the *X* direction. (a) Structure schematics of four configurations: convex, uniform, and two concave cases. (b) |*E*| field map at 5 mm from the structure. (c, d) |*H*| field maps that are parallel to the structure plane located 10 and 20 mm from the structure’s center, respectively. (e) 1D profile of the |*H*| field in the *X* direction at *Z* = 20 mm. (f) Full-width-half-maximum (fwhm) as a function of the four configurations. The black-dashed overlay shows the structure’s dimensions, and the red overlay shows the contour of half-maximum.

To examine nonuniform distribution of the subunits, configurations with a convex or concave distribution of the strips were designed. This was achieved by varying the distances between the strips in the *x* direction (Figure 3). The convex configuration featured a denser distribution of the strips in the center and the two concave ones, a denser distribution at the edges. The convex design can be utilized to condense the intensity of the magnetic field at the peak, while the concave ones disperse the intensity, thus providing larger coverage.

The design with short conducting strips and a nonuniform distribution both generated a high RF magnetic field intensity and provided extra control over the field coverage. Figure 3 shows the comparison of the uniform-strip distribution to the three nonuniform-strip configurations (one convex configuration and two concave setups). The 2D H-field maps and the 1D central line profile of the H-field show narrower H-field coverage with the convex setup and a wider coverage with the concave setups; these findings are summarized in the plot of the full-width-half-maximum (fwhm) of the H-field profile (Figure 3f). The H-field distribution at a distance of 20 mm and parallel to the structure showed a 10–15% increase in the fwhm with the concave configurations. Interestingly, one can notice partial smearing of the local peaks in the E-field maps in the concave configurations. However, the consequential heating should be carefully evaluated in the final setup, since it will also depend on the tissue properties in the region of interest.

### EM Simulations of the Full Setup for Brain Imaging at 7T MRI

To examine the advantages of the new metasurface in a realistic setup, a full setup simulation with a virtual human head model with a volume coil was performed by placing the structure in proximity to the temporal lobe.

**Figure 4:**
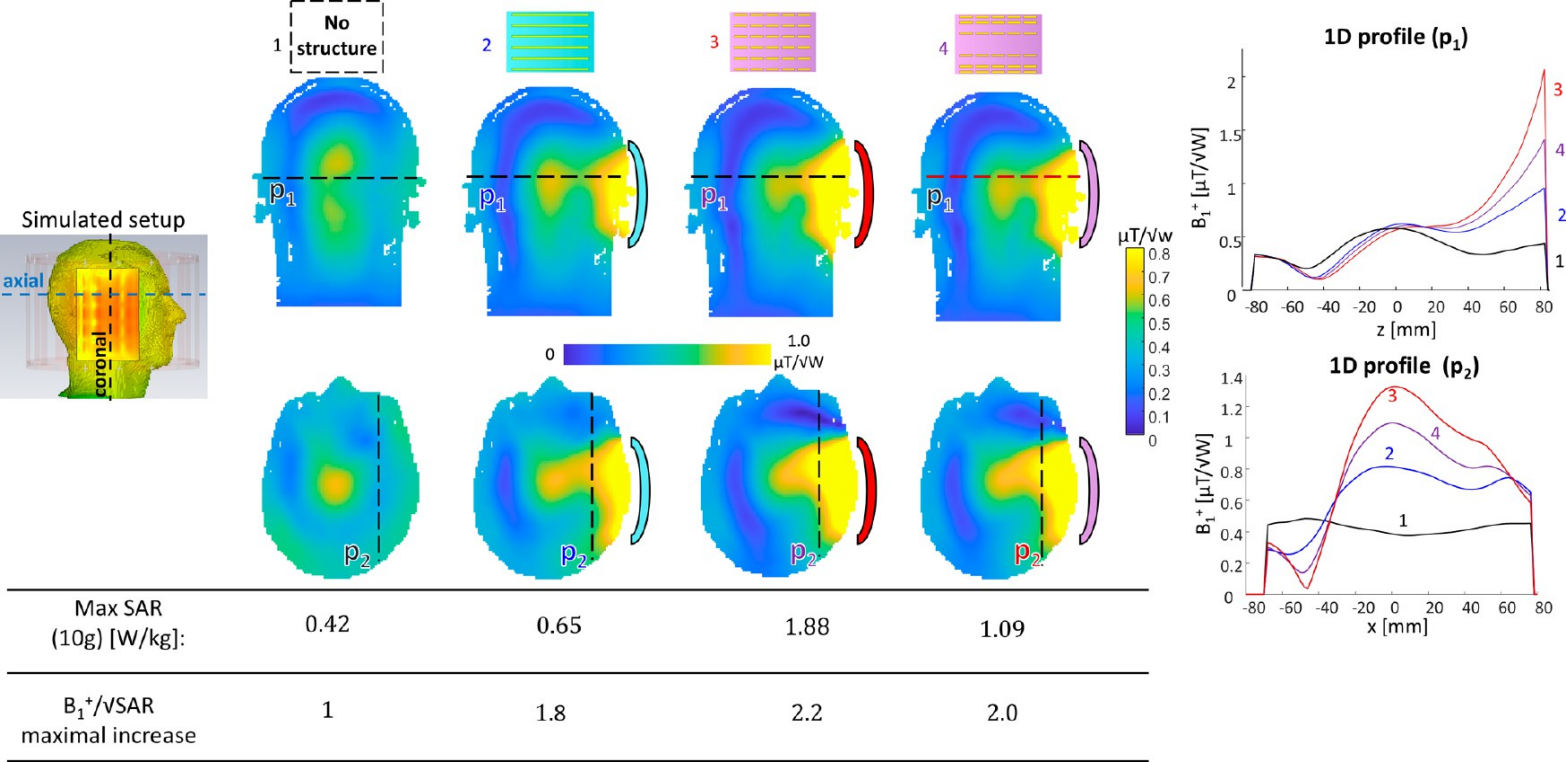
RF transmit field (*B*_1_^+^) maps of the EM simulation of the brain with added metasurface near the temporal lobe. From left to right: the setup, coronal (top), and axial (bottom) planes of the *B*_1_^+^ map without the added structure, with long-strips, with uniformly distributed short-strips, with a concave distribution of short-strips, and 1D profiles of the *B*_1_^+^ along the p1 and p2 lines (the lines are shown on the *B*_1_^+^ maps). The maximal specific absorption rate (SAR) and maximal RF transmit efficiency for each case are shown at the bottom.

A comparison of the achieved local magnetic field enhancement with the short-strip and long-strip designs was performed (Figure 4). The maximum enhancement of the RF magnetic field (*B*_1_^+^) with the added metamaterial was 4.7-fold and 2.2-fold higher with the short-strip and long-strip configurations, respectively, compared with the reference setup (without a metamaterial).

To examine the power deposition, the specific absorption rate (SAR) per 10 g was calculated. The SAR was significantly higher with the short-strip configuration. However, estimating the RF transmit efficiency, defined as (*B*_1_^+^/√SAR), resulted in a 2.2-fold and 1.8-fold increase with the short- and long-strip configurations, respectively, compared to the reference configuration.

When the concave configuration (no. 3 in Figure 3) was used, the SAR was reduced by a factor of 1.8 compared to the uniform distribution. In addition, the maximal B_1_ enhancement was 3-fold and the resulting RF transmit efficiency increased 2-fold compared to the reference configuration.

**Figure 5:**
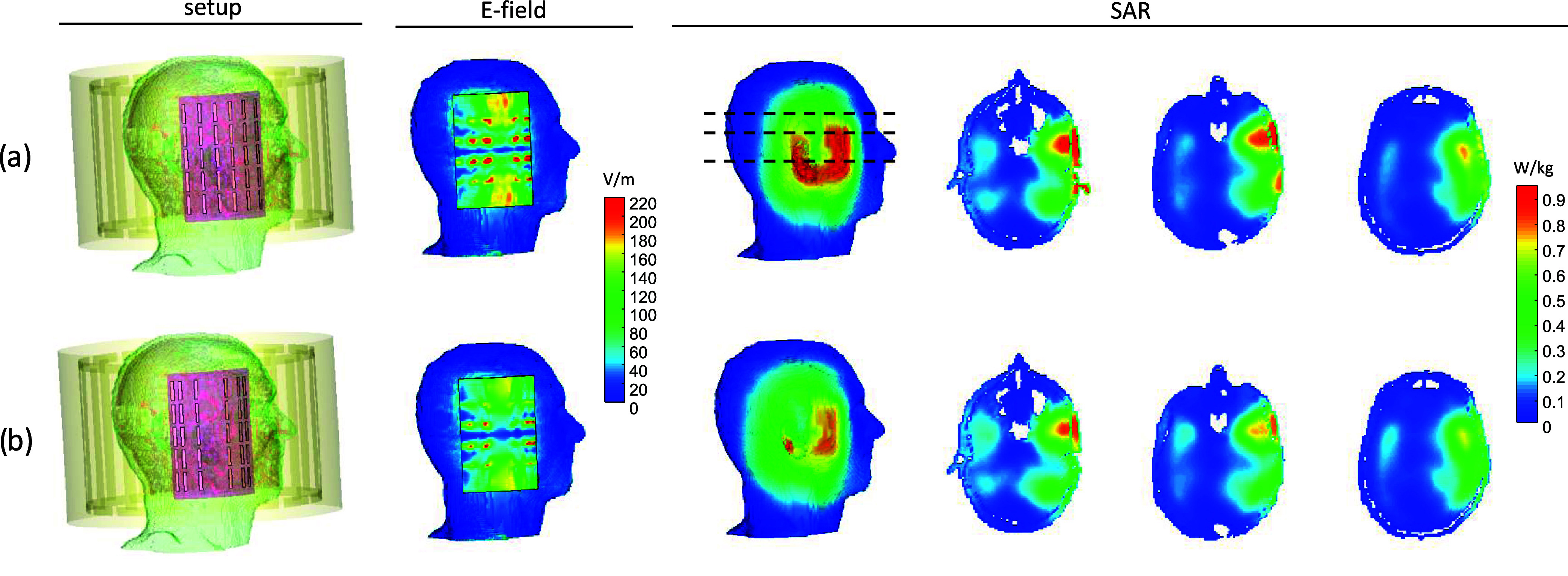
RF E-field and SAR maps of a full setup EM simulation of the brain with an added metasurface near the temporal lobe, comparing the uniform (a) and nonuniform (b) distribution of the short strips. From left to right: the setup, the E-field map on the metasurface plane, the SAR three-dimensional (3D) map in the brain, and three axial cross sections (the locations of the cross sections are shown with black-dashed lines).

A comparison of the electric field and SAR maps (Figure 5) demonstrated the advantage of the concave configuration over the uniform one; in addition to achieving a 1.8 factor reduction of the maximal SAR, it also shows reduced intensity at several local E-field hotspots. A similar set of EM simulations was also performed with the virtual human model Ella (see Supporting Information, S3). The B_1_ enhancement showed trends similar to those with the Duke model.

In addition, another brain imaging setup was examined, placing the metasurface near the occipital lobe (Supporting Information, Figure S4). This setup showed 3.5-, 2.7-, and 1.7-fold enhancement in the RF transmit efficiency with the uniform short-strip, concave short-strip, and uniform long-strip configurations compared to the reference configuration. In this setup, a 1.5-fold reduction in the SAR was achieved with the concave setup compared to the uniform setup.

**Figure 6:**
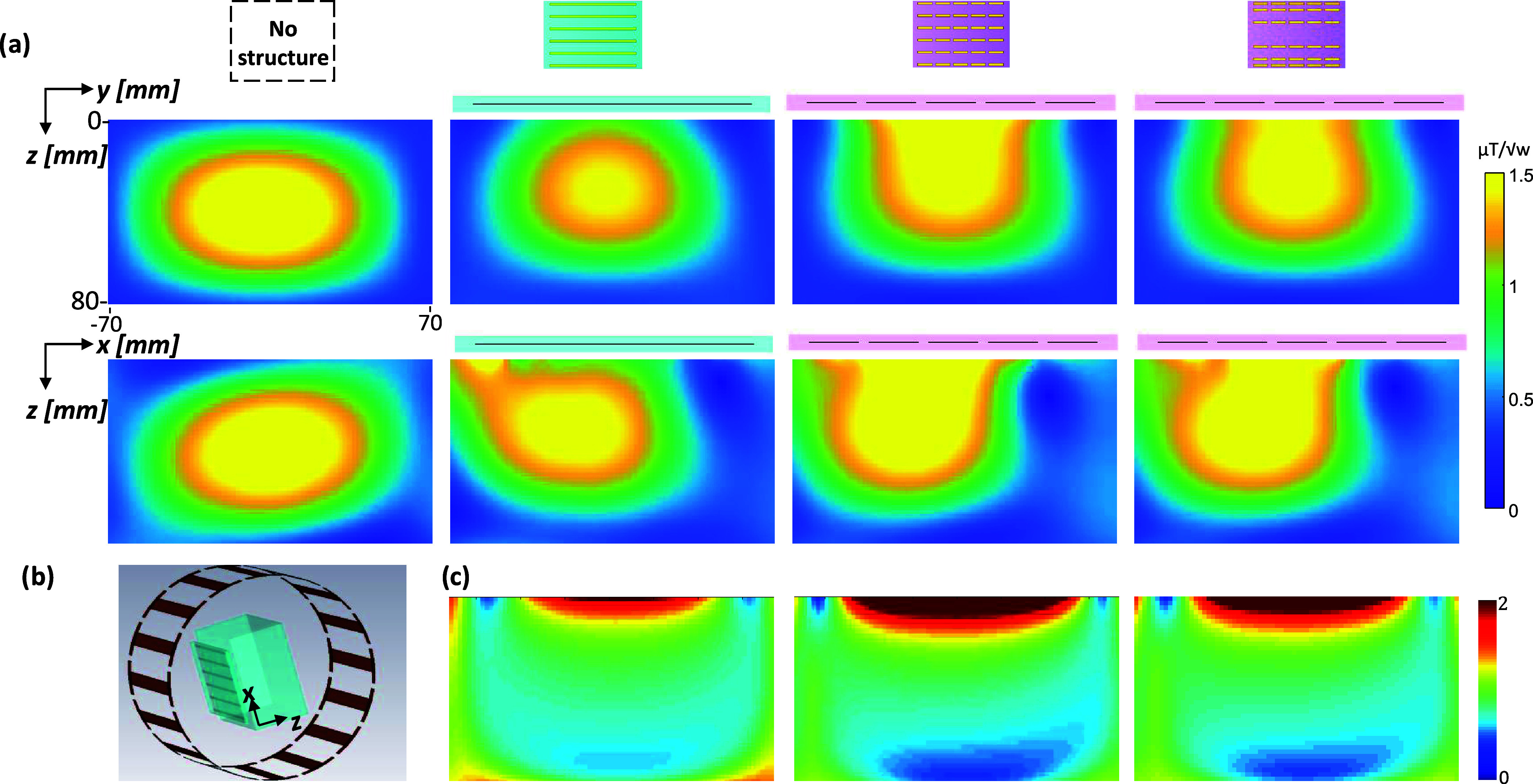
3D EM simulations of a phantom that mimics brain tissue. (a) *B*_1_^+^ maps for the different setups. From left to right: without any structure, used as a reference; with long-strip structure; with uniformly distributed short-strip structure; and with concave configuration of the short-strip structure. Top row shows *YZ* plane; mid row shows *XZ* plane (at the center of the phantom). (b) Schematics of the setup. (c) Ratio maps of the *B*_1_^+^ maps in the *YZ* plane divided by the reference shown in panel (a). The metasurface location is shown in the images (on top of the phantom).

**Figure 7:**
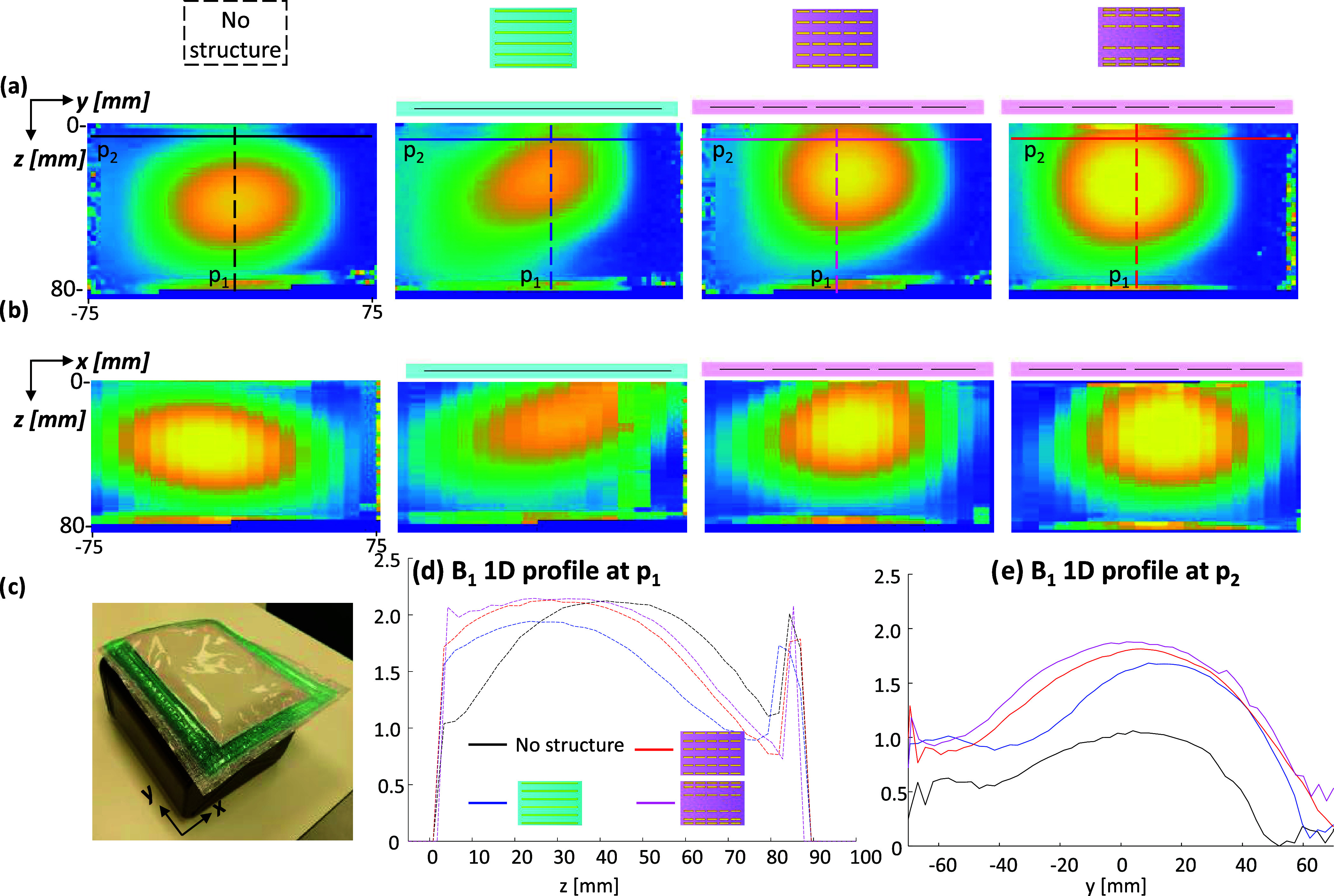
Comparison of the measured *B*_1_^+^ maps. (a) *B*_1_^+^ map at the *YZ* plane in the center of the phantom. (b) *B*_1_^+^ map at the *XY* plane at the center of the phantom. (c) Photo of the phantom with the added metasurface. (d, e) 1D profile of the *B*_1_^+^ along the p1 and p2 lines. The four configurations include a reference scan without an added metasurface, an added long-strip setup, a uniform distribution of the short-strips, and a concave distribution of the short-strips. The metasurface location is shown in the images (on top of the phantom).

Next, we fabricated short- and long-copper-strip setups positioned on a thin plastic substrate, which was then attached to the dielectric layer and sealed in a flexible plastic container (see Experimental Section for more details). Each construct was placed on top of a phantom that mimics brain tissue, and then 7T MRI scans were performed. Simulations of the same setups were also performed for comparison (Figure 6). The simulation results show the clear advantage of the new short-strip metasurface designs. The enhancement (measured 3 mm from the phantom edge) with the uniformly distributed short-strip configuration was 2.5-fold compared to a 1.6-fold enhancement with the uniformly distributed long-strip configuration. The short-strip setup with a concave distribution exhibited a slight reduction in the enhancement compared with the uniform short-strip setup (2.2-fold). The measured *B*_1_^+^ maps show slightly lower enhancements compared to simulation (Figure 7): 2.0-, 1.8-, and 1.6-fold enhancement with the uniformly distributed short-strip, concave distributed short-strip, and uniformly distributed long-strip configurations, respectively, compared to the reference (without added metasurface). Figure 7 shows a larger coverage in the *Z* and *Y* directions (in parallel to the structure and deeper into the phantom) by using the short-strip setup compared to the long-strip setup. Note that both simulations and measurements, in Figures 6 and 7, show an asymmetrical distribution of the *B*_1_^+^ field in the *XZ* plane (perpendicular to the main static field). This is an expected phenomenon at ultrahigh fields (explained in ref (25)), which is due to interference patterns at high fields and can be more pronounced in phantoms.

**Figure 8:**
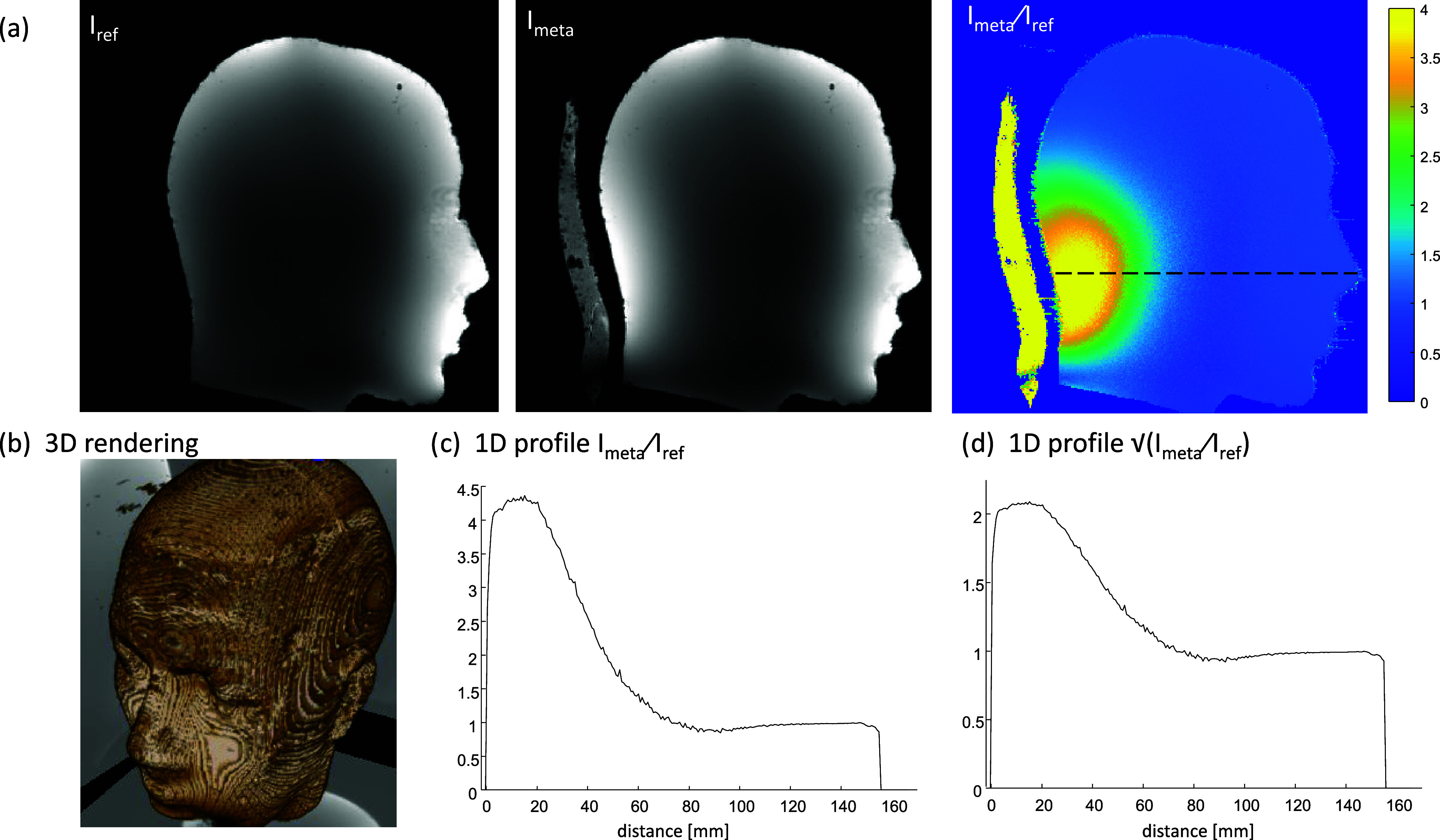
Sagittal MRI scan (gradient-echo) of a brain-mimicking phantom. (a) Images without (left) and with (middle) the added nonuniformly distributed short-strips metasurface (setup 3 in Figure 3). The map on the right shows the ratio between the two image intensities. (b) 3D rendering of the brain-mimicking phantom. (c) 1D profile of the ratio *I*_meta_/*I*_ref_ (*I*_meta_—image with the metasurface, *I*_ref_—reference image without the metasurface). The line of the plot is shown as a dashed line on the ratio map in panel (a). (d) 1D profile plot of the √*I*_meta_/*I*_ref_ (which is proportional to the *B*_1_^+^ increase) for the same dashed line.

In addition, an MRI scan (a low flip angle gradient-echo) without and with the nonuniformly distributed short-strips metasurface was performed with a brain-mimicking phantom. This is another method to examine the increase in *B*_1_^+^, since the SNR of the image in such a scan is proportional to sin(γ*B*_1_^+^τ)·(*B*_1_^–^*)/√*P*, which can be approximated as γ(*B*_1_^+^)^2^τ·/√*P* in the low flip angles regime (γ is the gyromagnetic ratio, τ is the pulse duration, and *P* is the accepted power of the coil. *B*_1_^–^* is the conjugate of the receive field *B*_1_^–^ that is defined as (*B*_1*x*_ – *iB*_1*y*_)/2). Thus, taking the square root of the ratio between the images (*I*_meta_/*I*_ref_, *I*_meta_—image with metasurface, *I*_ref_—image without the metasurface), one can estimate the *B*_1_^+^ increase. Figure 8 shows the results with a 2-fold enhancement in the √*I*_meta_/*I*_ref_. The details of the brain-mimicking phantom and the scan are included in the Experimental Section.

## Discussion and Conclusions

This study examined a new MRI-viable metasurface based on a 2D array of short conducting strips. The new design was used to produce a resonant structure capable of increasing the local RF transmit field in 7T MRI. An MRI-viable resonant assembly was achieved by adding a high dielectric material (using a relative permittivity of 160–164). We demonstrated that this new short-strip design, when placed in proximity to the temporal lobe region, significantly increases the RF local transmit field in a brain imaging setup: 4.7-fold increase using a short-strip design vs a 2.2-fold increase using a long-strip design, both relative to the setup without a metamaterial.

However, it comes at the expense of the SAR, which increased in the short-strip configuration. Yet, the estimated RF transmit efficiency with the short-strip configuration was still higher than with the long-strip configuration (2.2-fold vs 1.8-fold, respectively). Another set of simulations, placing the metasurface near the occipital lobe, showed a similar trend, with superior results using the short-strip configuration compared with the long-strip configuration.

To deal with the increased SAR, this study investigated a nonuniform distribution of the strips as another tool to shape both the RF magnetic and electric field distributions. As expected, the convex distribution can be used to increase the maximal peak of the H-field (by focusing the intensity of the magnetic field at the peak, similar to a convex lens), while the concave distribution provides wider H-field coverage (by 10–15%) and reduces the intensity of the H-field peak (by dispersing the intensity). Another advantage of the concave distribution over the uniform one was 1.5- to 1.8-fold SAR reduction while preserving most of the RF transmit field. The reduction in SAR can be attributed to the increased density of the conducting strips at the edges, which locally reduces the electric field where it is highest.

The dielectric substrate used in this structure implementation was based on a CaTiO_3_ and BaTiO_3_ suspension, which offers the advantage of a flexible cushion-like implementation. However, it can also result in increased losses. Rigid ceramics can also be used to realize the required dielectric properties.^26,27^ Of note, a recent work introduced an artificial dielectric implementation in MRI,^28^ which could retard the need for a dielectric substrate.

In summary, this study demonstrated the potential of short-strip metasurface in 7T MRI to improve the local RF transmit field, while emphasizing the importance of the spatial geometry of the subunits in the resulting power deposition. Next-generation RF coils can benefit from metamaterial concepts while taking into account the constraints of the patient environment.

## Experimental Section

### Characterization of the Metasurface Structure

The eigen-mode solver and full setup EM simulations were performed by using CST Studio Suite 2019 (Dassault Systemes Deutschland GmbH). The assemblies of the array of the conducting short strips and long strips were tuned by varying the dielectric substrate’s relative permittivity to reach the TE_01_ resonant mode at 298 MHz. The total dimensions of all setups were the same: 16 × 11 × 0.7 cm^3^ (length, width, and thickness, respectively). The details of the two setups were:

(a) Short-strip uniform configuration: A matrix of 6 × 5 short copper strips, each 20 mm in length, representing magnetic dipoles, equidistantly spaced 20 mm apart in *X* direction and 5 mm in *Y* direction (see Figure 1), and with a dielectric layer of ε_r_ = 164 (Figure 1a).
(b) Long-strip uniform configuration: An array of 6 uniformly spaced 140 mm long copper strips, representing electric dipoles, equidistantly spaced 20 mm apart, and with a dielectric layer of ε_r_ = 72 (Figure 1b).

To examine the nonuniform distribution, 4 short-strip setups were compared (see Figure 3a):

I. Short-strip convex configuration (case #1): 6 × 5 matrix of short copper strips, each 20 mm in length, with the following five spacing in the *X* direction: 30, 15, 10, 15, 30 mm. A dielectric layer of ε_r_ = 170 was used.
II. Short-strip uniform configuration (case #2): The same as in (a) above.
III. Short-strip concave configuration (case #3): 6 × 5 matrix of short copper strips, each 20 mm length, with the following five spacing in the *X* direction: 10, 20, 40, 20, 10 mm. A dielectric layer of ε_r_ = 160 was used.
IV. Short-strip concave configuration (case #4): 6 × 5 matrix of short copper strips, each 20 mm in length, with the following five spacing in the *X* direction: 7, 13.5, 60, 13.5, 7 mm. A dielectric layer of ε_r_ = 160 was used.

3D EM simulations of the *B*_1_^+^ field were performed using FIT (finite integration technique) and CST software. All *B*_1_^+^ maps were normalized to an accepted power of 1 W. The simulation setup included a 16-rung high-pass quadrature birdcage coil (inner diameter of 30 cm, rung length of 18 cm). The “Duke” and “Ella” human models from the Virtual family using a mesh resolution of 1 × 1 × 1 mm^3^ were used to simulate the RF transmit field in the head region of interest. The metasurface structures were added in the proximity of the temporal lobe as shown in Figures 4 and 5 and in the proximity of the occipital lobe as shown in Supporting Information, Figure S4. The metamaterial-based structure was curved to best fit the shape of the head.

In addition, a full setup of EM simulations with a rectangular phantom that mimics brain tissue was performed to enable a comparison to the measured results. The phantom electrical properties were ε_r_ = 53 and conductivity (σ) = 0.3 S/m. The phantom’s size was 14 × 8 × 16 cm^3^.

The axes convention we choose for all figures is that the *Z*-axis is perpendicular to the structure, the *X*-axis is along the shorter dimension of the metasurface, and the *Y*-axis is along the longer direction of the metasurface. The same axes were also used in the B_1_ maps for consistency.

### MRI Measurements and Metasurface Implementation

To implement the dielectric layer of the short-strip setup, a suspension of BaTiO_3_, CaTiO_3_, and water was used; 5.1:3:1 ratio for ε_r_ = 164 and 5.1:2.7:1 for ε_r_ = 160. The permittivity for the long-strip setup was implemented using water only, while the water volume was tuned to tune the structure resonance at 298 MHz. The phantom container was filled with sucrose-water suppression with 52% sucrose and 0.5% NaCl to achieve ε_r_ = 53 and σ = 0.3 S/m. The short-strip design was implemented with the uniform (case #2) and concave (case #3) configurations.

The metasurface was placed on top of the phantom and scanned in a 7T MRI (MAGNETOM Terra, Siemens Healthcare, Erlangen) with a 1Tx/32Rx Nova coil. Scans using the vendor’s B_1_ map (based on a preconditioning RF pulse with a Turbo FLASH readout^29^) sequence were collected using a 20 × 20 cm^2^ FOV and a spatial resolution of 2.5 × 2.5 × 3.5 mm^3^.

The brain-mimicking phantom that was used here is based on a head-shaped container filled with an fBIRN^30^ recipe (designed for 7T brain imaging), giving electrical conductivity and T_1_ relaxation time properties similar to the brain tissue. Scan parameters were low-flip-angle 3D gradient-echo sequence with field-of-view (FOV) = 22 × 22 × 12 cm^3^, spatial resolution = 0.7 × 0.7 × 2.0 mm^3^, TR/TE = 7/3.14 ms, and flip angle = 5°.

## Supporting Information Available

Comparison of surface current distribution, between the new design and a setup based on long strips; three metasurface designs of different dimensions, all resonant at 298 MHz; additional human brain model EM simulations, examining additional metasurface locations and an additional human model; vector plots of the surface currents (PDF)
