# Supplementary material for: Shaping the RF Transmit Field in 7T MRI Using a Nonuniform Metasurface Constructed of Short Conducting Strips
Source: ACS Appl Mater Interfaces. 2024 Aug 31;16(36):47284–93. doi: 10.1021/acsami.4c10402 (PMC11403565; doi:10.1021/acsami.4c10402)
Supplement: Supplementary file 1 — am4c10402_si_001.pdf [file am4c10402_si_001.pdf]

Supporting Information:

Shaping the RF transmit field in 7T MRI using a non-uniform metasurface constructed of short conducting strips

Santosh Kumar Maurya[1,2] *and Rita Schmidt*[1,2]*

[1]Department of Brain Sciences, Weizmann Institute of Science, Rehovot, postal code 7610001, Israel

[2]The Azrieli National Institute for Human Brain Imaging and Research, Weizmann Institute of Science, Rehovot, postal code 7610001, Israel

*E-mail: rita.schmidt@weizmann.ac.il

S1. Surface currents distribution

In this study, a metasurface based on an array of short conducting strips with high dielectric substrate was designed. A comparison of the surface current distribution in this setup and a setup based on a set of long strips was performed. A vector plot of the surface currents is shown in Figure S1. The short-strip setup shows loop-like circuits that are characteristic of magnetic-dipole behavior, which are not apparent in the long-strip setup. Note, that due to the finite number of rows and columns in this implementation, this design has both magnetic dipole and electric dipole components, but magnetic-dipole one is dominant in the center.

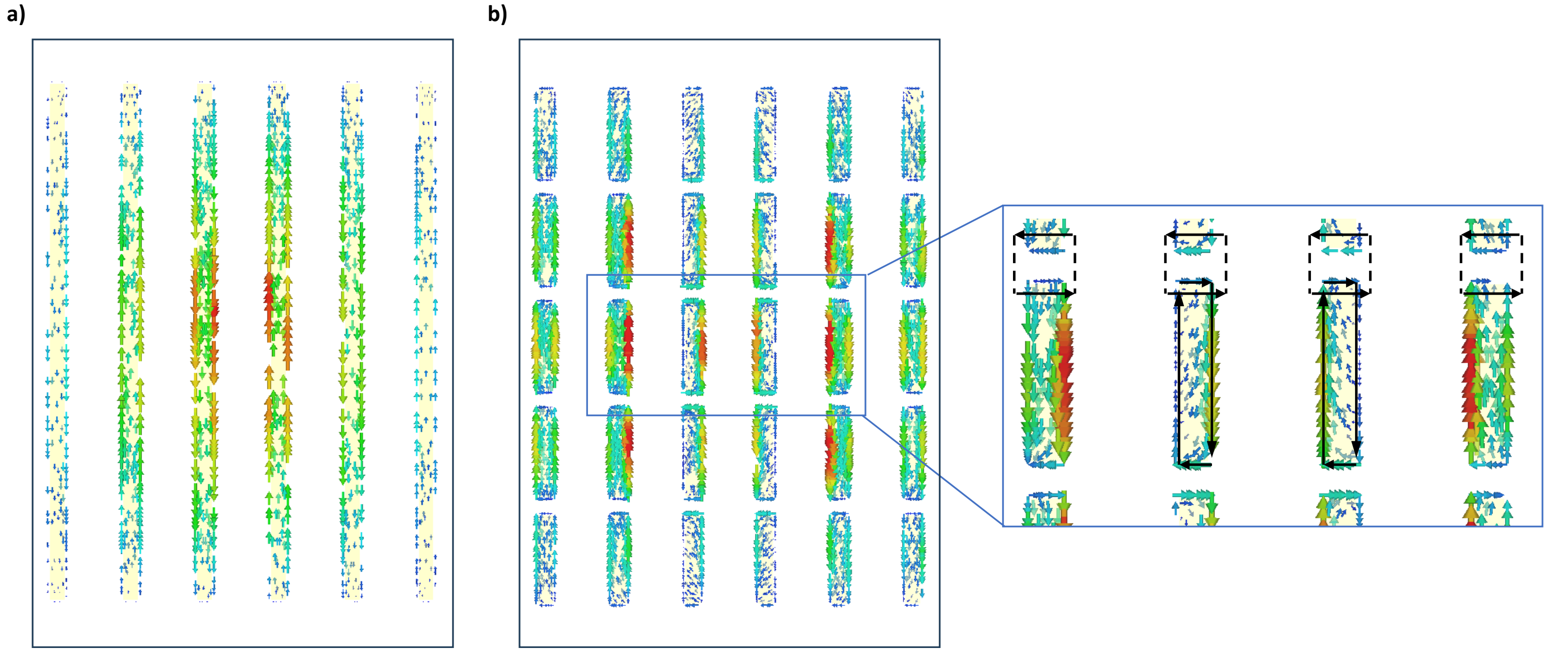

Figure S1. Vector plots of the surface currents. a) long-strips setup, b) short-strip setup. The short-strips setup shows zoom-in with assemblies of loop circuits that are characteristic of magnetic-dipole behaviour, which are not apparent in the long-strips setup.

S2. Resonant mode at 298 MHz with three different dimensions

In this study, a setup for brain imaging was optimized with overall dimensions of 16x11x0.7 cm^3. However, having other applications in mind, a resonant mode at 298 MHz with other dimensions can be achieved as well. Here, three setups are shown, the one that was used in this study and two additional configurations, one of a smaller size (a square setup – 14x14 cm^2) and one of a larger size, 18x15 cm^2. We varied the thickness of the dielectric substrate to tune the TE_{01} mode to 298 MHz. Figure S2 shows the resulting H-field of the three configurations. A metasurface with smaller dimensions has higher maximal intensity, but smaller penetration depth, while larger dimensions has lower

maximal intensity, but larger penetration depth. The dimensions can be chosen, depending on the particular application in mind.

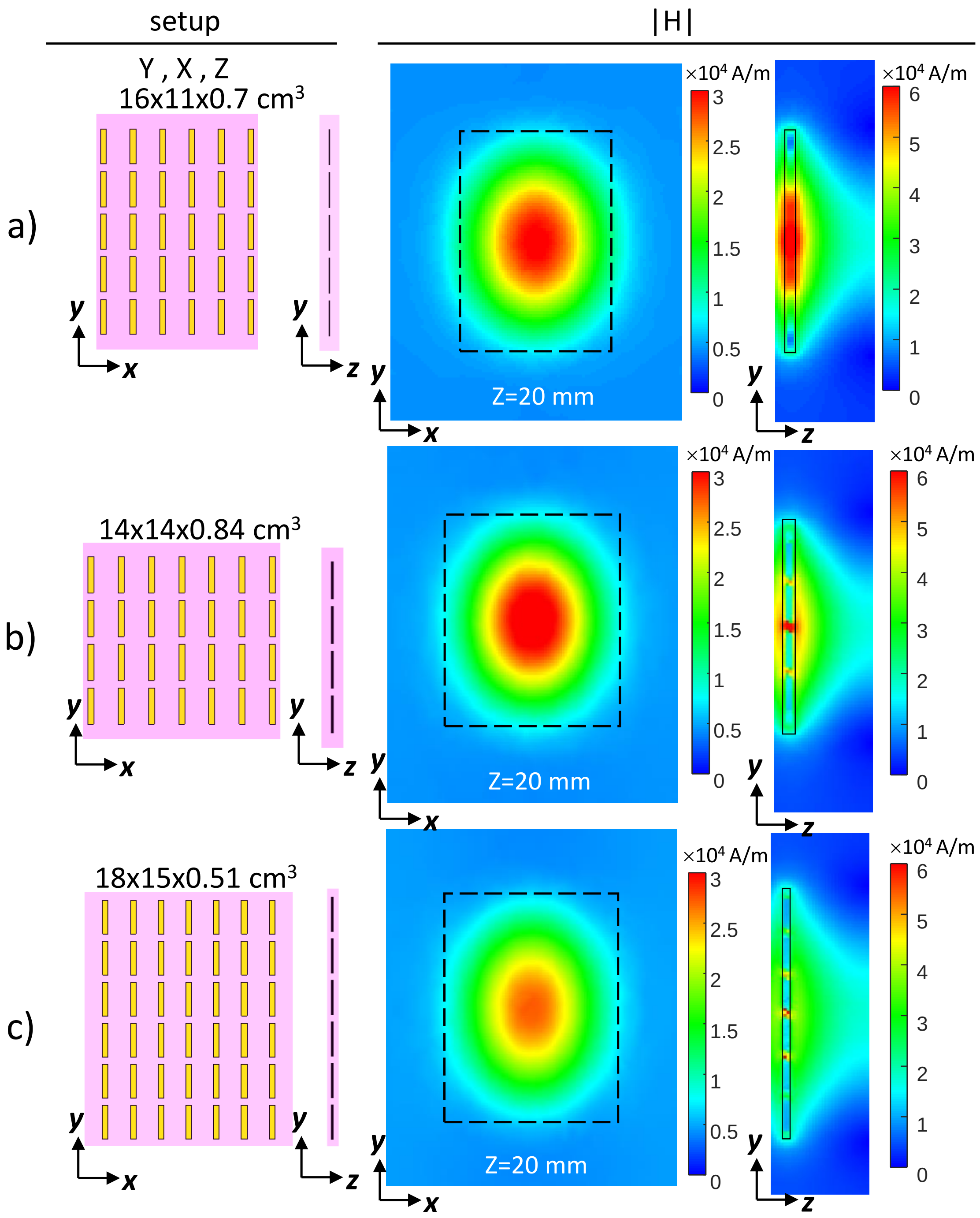

Figure S2. Resonant TE_{01} mode at 298 MHz for three setups with three different dimensions. a) 16x11x0.7 cm^3, b) 14x14x0.84 cm^3, a) 18x15x0.51 cm^3. From left to right – the setup, the H-field at Z=20 mm from the structure and H-field perpendicular to the structure. The dashed-line shows the metasurface dimensions on the H-field maps. The relative permittivity of 164 was used for all setups.

S3. 3D EM simulations with human brain model

In addition to the Duke human model, Ella model was simulated with a similar setup to the one shown in Figure 4 (including a volume coil), where the metasurface is in the proximity to the temporal lobe. Figure S3 summarizes the results with similar trends to the simulations with Duke model. B_1^+ maximal increase was 2-fold compared to a reference with long-strip, 3.3-fold increase with uniform short-strip and 2.5-fold with non-uniform short-strip setups. SAR is increased with the uniform short-strips setup compared to the long-strip. The non-uniform distribution reduced the SAR compared to the uniform distribution setup.

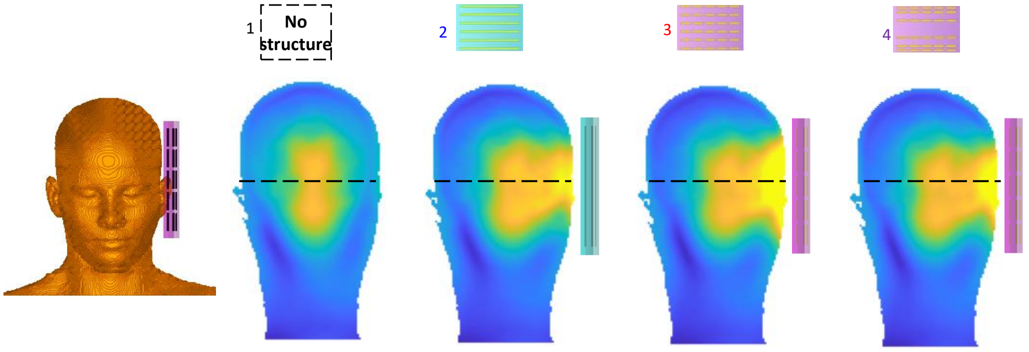

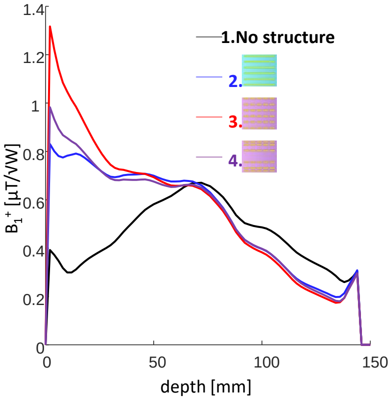

Max SAR (10g) [W/kg]:	0.59	1.50	1.84	1.60
B_1^+[µT/√w] maximal increase	0.4	0.83	1.32	1.0

Figure S3. RF transmit field (B_1^+) maps of the EM simulation with Ella brain model with added metasurface near the temporal lobe. From left to right: the setup, coronal plane of the B_1^+ map without the added structure, with long-strip, with uniformly distributed short-strip, with a concave distribution of short-strip configurations, and 1D profile of the B_1^+ as function of the depth in the tissue (see black dashed line on the B_1^+ maps). The maximal SAR and maximal B_1^+ for each case are shown at the bottom.

Additional brain imaging full setup EM simulation was performed with the Duke virtual human head model with a volume coil and placing the metasurface near the occipital lobe. This setup showed 3.5-, 2.7- and 1.7-fold enhancement in the RF transmit efficiency with the uniform short-strip, concave short-strip and uniform long strip configurations

compared to the reference configuration. In this setup a 1.5-fold reduction in the SAR was achieved with the concave setup compared to the uniform setup. See Figure S4 below:

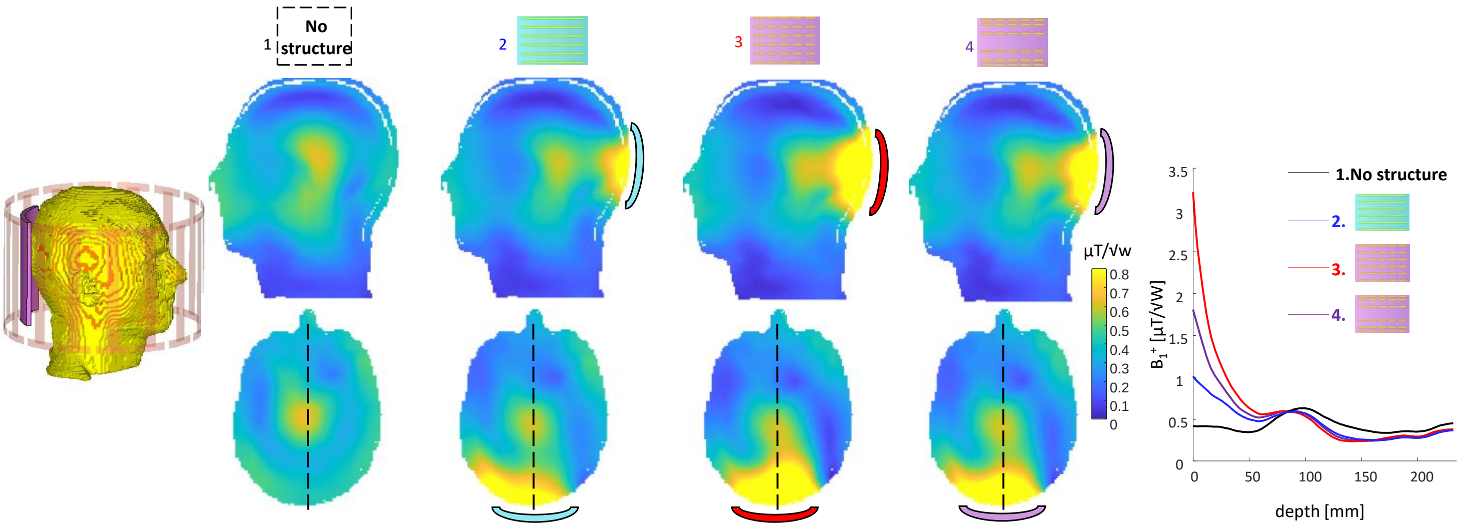

Max SAR (10g) [W/kg]:	0.42	0.78	1.24	0.84
B_1^+[μT/√w] maximal increase	1	1.7	3.5	2.7

Figure S4. RF transmit field (B_1^+) maps of the EM simulation of the brain with added metasurface near the occipital lobe. From left to right: the setup, sagittal (top) and axial (bottom) planes of the B_1^+ map without the added structure, with long-strip, with uniformly distributed short-strip, with a concave distribution of short-strip configurations, and 1D profile of the B_1^+ as function of the depth in the tissue (see black dashed line on the B_1^+ maps). The maximal SAR and maximal B_1^+ for each case are shown at the bottom.
